# Direct measurement of the genuine efficiency of thermogalvanic heat-to-electricity conversion in thermocells†

**DOI:** 10.1039/d1sc06340e

**Published:** 2022-04-05

**Authors:** Maria A. Trosheva, Mark A. Buckingham, Leigh Aldous

**Affiliations:** Department of Chemistry, King's College London Britannia House London SE1 1DB UK leigh.aldous@kcl.ac.uk

## Abstract

Harvesting wasted thermal energy could make important contributions to global energy sustainability. Thermogalvanic devices are simple, chemistry-based devices which can convert heat to electricity, through facile redox chemistry. The efficiency of this process is the ratio of electrical energy generated by the cell (in Watts) to the quantity of thermal energy that passes through the cell (also in Watts). Prior work estimated the quantity of thermal energy passed through a thermocell by applying a conductive heat transfer model to the electrolyte. Here, we employ a heat flux sensor to unambiguously quantify both heat flux and electrical power. By evaluating the effect of electrode separation, temperature difference and gelation of the electrolyte, we found significant discrepancy between the estimated model and the quantified reality. For electrode separation, the trend between estimated and measured efficiency went in opposite directions; as a function of temperature difference, they demonstrated the same trend, but estimated values were significantly higher. This was due to significant additional convection and radiation contributions to the heat flux. Conversely, gelled electrolytes were able to suppress heat flux mechanisms and achieve experimentally determined efficiency values in excess of the estimated values (at small electrode separations), with partially gelled systems being particularly effective. This study provides the ability to unambiguously benchmark and assess the absolute efficiency and Carnot efficiency of thermogalvanic electrolytes and even the whole thermocell device, allowing ‘total device efficiency’ to be quantified. The deviation between the routinely applied estimation methodology and actual measurement will support the rational development of novel thermal energy harvesting chemistries, materials and devices.

## Abstract 2

We report the first genuine quantification of thermogalvanic heat-to-electricity conversion efficiency, for both the electrolyte and for the entire device.

## Graphical abstract

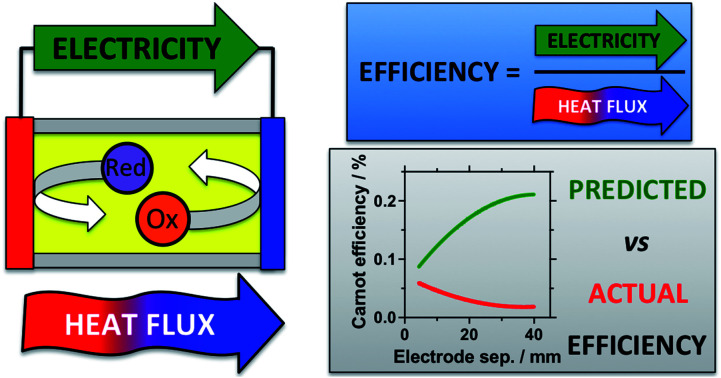

## Introduction

Thermoelectrochemistry – or electrochemistry where temperature is applied as an active variable – has many implications and applications, given the significant impact of temperature upon both thermodynamic and kinetic parameters.^1^ A key thermodynamic parameter is the temperature effect upon the standard electrode potential.^2,3^ A growing application of this is thermogalvanic cells; these are devices that typically comprise of two electrodes at different temperatures in contact with a solution or gel that contains both oxidation states of the same redox couple (*i.e.* both [Fe(CN)_6_]^3−^ and [Fe(CN)_6_]^4−^ or Fe^2+^ and Fe^3+^).^4^ The presence of a temperature difference (Δ*T*) across the two electrodes results in a thermodynamically-driven induced potential difference (Δ*V*) between the electrodes, which drives redox processes that results in a flow of electrical current. This, coupled with diffusion of the redox couple between the two electrodes – results in an elegant, purely chemical route for the conversion of a temperature gradient into electricity.^5^ Given that *ca.* two-thirds of the energy from human industry (even down to the human metabolism) is dissipated as low grade waste heat,^6^ widespread application could result in major efficiency gains, *e.g.* such as on industrial piping^7^ or skin.^8–10^

Thermogalvanic electricity production is an entropically-driven process, and the magnitude of the driving force is normally expressed as the ‘thermogalvanic Seebeck coefficient’, or *S*_e_;1
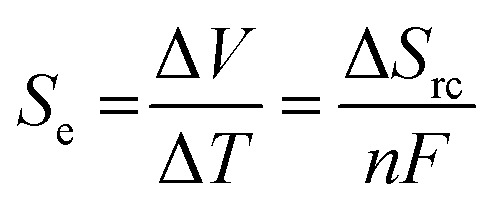
where Δ*S*_rc_ is the difference in entropy between the two redox states, *n* is the number of electrons transferred and *F* is the Faraday constant.^11^ The *S*_e_ therefore represents the possible potential difference; Soret effects^12^ and thermocapacitive effects^13,14^ can also be significant contributors to the potential difference, but are only significant in fairly unique (typically gelled) systems. The *S*_e_ is sensitive to the absolute concentration of the redox couple, the ratio of the concentration of the two oxidation states, and the ionic strength.^4^ The current generated by this potential difference is also sensitive to the concentration and ratio of the redox species,^4^ and especially sensitive to the *S*_e_ and Δ*T*;^11^ it's also sensitive to the mass transport of the two redox states (diffusion coefficient, convection, inter-electrode transport distance, *etc.*), the kinetics of the redox process, and the electrode surface area.^11^

**Fig. 1:**
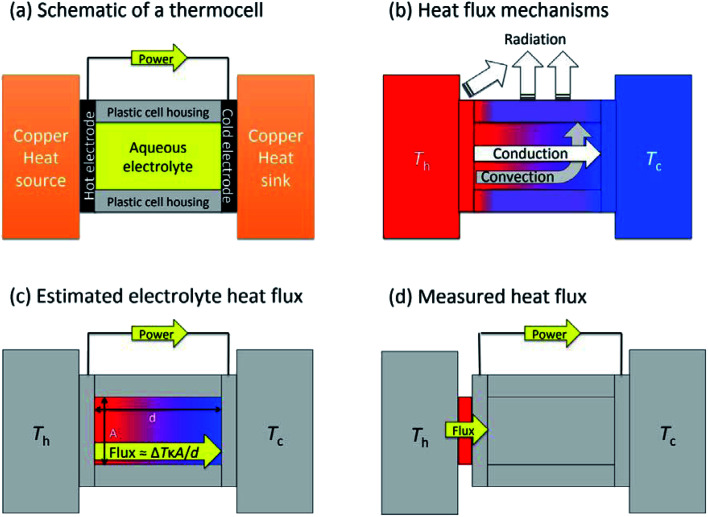
Diagrams highlighting (a) the general layout of the thermogalvanic cell employed here, whereby electrical power can be generated (and measured) when a temperature difference is applied *via* the heat source/sinks; (b) the three heat flux mechanisms expected (if the temperature of the heat source, *T*_h_, is significantly above *T*_c_, and *T*_c_ is close to the ambient temperature). This figure excludes air-convection and also the metallic wiring, which can be an additional source of conduction; (c) how the heat flux is typically estimated, using an electrolyte-only, conduction-only approximation; and (d) the experimental design employed in this study whereby all heat flux through the heat source into the cell is quantified simultaneous to electrical power quantification.

Fig. 1(a) shows a general layout for measuring a thermogalvanic cell, where a temperature difference can be easily applied across the two electrodes. The electrical power produced by the two electrodes can then be easily quantified *via* a variety of routes.^15^ Once the cell comes to equilibrium and steady state power output is achieved, the power is often limited by one dominant resistance, which is typically either kinetics (*e.g.* rate of electron transfer) or the rate of mass-transport between the two electrodes.^5,15^ This one major resistance factor, *R*, means the thermogalvanic cell displays a linear but inverse relationship between the voltage and the current produced, in line with Ohm's Law, so *V* = *IR*. The maximum voltage from the cell is characterised as the open circuit potential (*V*_ocp_, in V), and the maximum current as the short-circuit current density (*j*_sc_, in A m^−2^). Power generated follows Watt's Law, so *P* = *IV*. The inverse relationship between *V*_ocp_ and *j*_sc_ means that thermogalvanic cells typically generate a parabolic power curve,^15^ where the maximum power density generated by the cell (*P*_max_, W m^−2^) occurs at half the maximum current and half the maximum voltage, such that;

2 *P*_max_ = 0.25*V*_ocp_*j*_sc_

Thermogalvanic conversion of thermal energy to electrical energy is not a perfect process and is limited by both Carnot efficiency and non-ideal processes. Fig. 1(b) highlights the scenario if the hot electrode temperature is far above ambient temperature while the cold electrode is close to ambient; conduction of heat will occur through the cell and electrolyte, temperature-difference induced convection in the electrolyte will exacerbate this, and radiative heat loss into the surroundings will also occur. The thermoelectric-inspired ‘dimensionless *ZT* figure of merit’ is sometimes referred to in thermogalvanic literature in an effort to consider the competing thermogalvanic current and heat flux processes;3
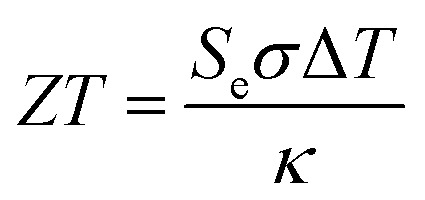
where the bigger the *ZT* the more ‘merit’ the device has for power production, with *σ* the ionic conductivity of the electrolyte and *κ* the thermal conductivity (steady state, conduction-only, and in the absence of a temperature gradient). This approximate relationship is largely valid for thermoelectrics, since the entire device is typically solid, and electrical conductivity correlates well with charge carrier mobility. However, this is a flawed metric for thermogalvanic systems, because the *σ* typically significantly overestimates the quantity and mobility of the actual charge carriers in the cell because it measures all ions including non-redox-active supporting electrolyte,^16^ while the *κ* significantly underestimates heat transfer through a mobile convection-prone liquid exposed to a temperature difference.^7^

The absolute efficiency, *η*, is another key (and in theory unambiguous) means of benchmarking thermogalvanic device performance. The absolute efficiency is quantified by the proportion of thermogalvanic electrical power generated by the cell (*p*_max_ in W) from the corresponding heat flux passing into the cell (*q*, also in W), as shown in eqn (4). This can then be expressed as the fraction of the theoretically limiting Carnot cycle efficiency, *η*_r_, based upon the applied hot and cold electrode temperatures (*T*_h_ and *T*_c_, respectively), as shown in eqn (5);4
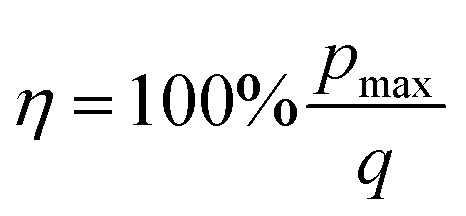
5
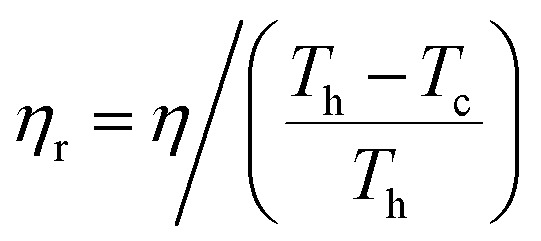


Arguably the magnitude of the efficiency doesn't necessarily matter; if thermal energy that would otherwise be wasted is instead valorised then that can be a net benefit. It's been stated that thermogalvanic devices with Carnot-relative efficiencies in the range of 2 to 5% could be commercially competitive,^11^ although a full technoeconomic comparison is still required to confirm if these values should be higher or lower. Nevertheless, the more efficient this process is at valorising waste, the more ‘green’ it is (alongside a series of other considerations).^17,18^ Additionally the more green and cost-effective it is, the more ‘sustainable’ the process is.^19^

In an effort to benchmark efficiencies, an ‘electrolyte-only’ heat flux estimation is commonly employed for thermogalvanic cells. This is exemplified in Fig. 1(c). Typically, the heat flux through the cell housing itself is disregarded, since most early cell constructions were not representative of application-appropriate cells. Instead, only the body of the electrolyte is considered, and is treated as a 1D heat flux through a 2D solid material using Fourier's Law;6
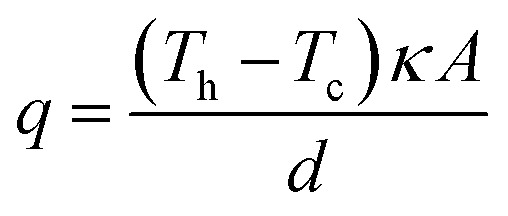
where *κ* is the (steady state, conduction-only) thermal conductivity of the electrolyte again, *A* the cross-sectional area of the electrolyte, and *d* the distance between the two electrodes.

Early adoption of this estimation was mathematically justified, since it was suggested convective heat flux was negligible under their particular circumstances.^11^ However, cell design has since diversified, and several studies have highlighted the very significant role convection can play in certain thermogalvanic cells.^20–22^ The use of *IR* cameras in electrolyte measurements explicitly relies upon radiation out of the cell,^20,23^ which is also not considered by eqn (6). Numerous application-appropriate cell designs have also been reported, such as integrated into clothing,^8^ wearable on skin,^9,10^ installed onto hot water pipes,^7^*etc.*, yet heat flux quantification has not advanced in step with this progress. While flawed, this estimated heat flux has nevertheless been the most valid approach available for benchmarking the efficiencies of new systems, with several solution-based, nano-structured electrode systems having Carnot-relative efficiencies predicted in excess of 0.5%,^7,23–33^ and even >2%.^23,34–36^

Two recent studies have gone beyond directly measuring thermogalvanic power and estimating the heat flux. Wu *et al.* investigated gelled *vs.* non-gelled electrolytes, and calculated the efficiency *via* the estimated heat flux; no significant difference was observed between the two types of electrolytes.^22^ However, the cold electrode temperature was maintained using a peltier device, and the power required to keep that electrode isothermal was two-fold higher in the ungelled electrolyte than the gelled electrolyte, demonstrating there was a significant difference in the genuine bulk heat flux through the cell that was not detected by the thermogalvanic measurements; likely because the thermal apparatus actively compensated for this difference by providing more cooling power.^22^ Yu *et al.* reported the efficiency of a thermogalvanic cell using the estimated heat flux equation, but rather than apply the conduction-only thermal conductivity, *κ*, they used *IR* imaging to estimate the effective thermal conductivity, *κ*_eff_.^23^ This indirect measurement (*via* heat radiated from a cell wall) suggested that at an applied temperature difference of 50 K, the *κ*_eff_ reached 1.64 W m^−1^ K^−1^;^23^ far above the *κ* = 0.55 W m^−1^ K^−1^ measured in other studies.^34,37^ Interestingly, the addition of guanidinium resulted in crystallisation of the [Fe(CN)_6_]^4−^, and the presence of this solid material in the cell reduced *κ*_eff_ to *ca.* 0.4 W m^−1^ K^−1^. This value of *κ*_eff_, combined with a significantly boosted *S*_e_ value due to the crystallisation process, resulted in a very significant electrolyte-only Carnot efficiency value of 11.1% being stated (at Δ*T* = 40 K).^23^

The purpose of this study was to prepare a thermoelectrochemical cell that could unambiguously measure both the heat flux and the thermogalvanic power; the ratio of these two quantified values will then yield the absolute efficiency for an entire thermogalvanic device, for the first time. Fig. 1(d) describes this concept, whereby a heat flux sensor is introduced as a thermal bottleneck through which all thermal energy from the hot electrode must pass, thus allowing total quantification of the heat flux (*q*_total_, which subsequently passes through and out of the cell, *via* conduction, convection and/or radiation). If the heat flux through the empty cell, *q*_empty_, is subtracted it can yield a directly measured and quantified electrolyte-only heat flux, *q*_m_. That was performed in this study, and the trends in efficiency compared between those obtained using direct measurements *vs.* those predicted using the estimated heat flux given *via*eqn (6). This study was used to achieve unambiguous quantification, then explore the effect of the parameters Δ*T*, *d* and gelled *vs.* un-gelled electrolytes upon efficiency.

## Experimental

### Electrode material comparison using conventional thermogalvanic assembly

Initial experiments used a setup that has been previously reported in detail elsewhere;^4^ namely a 6.7 mm diameter cylinder was machined from a block of PMMA (8.4 mm deep). At either end larger, shallow cylinders (10 mm diameter, 0.5 mm deep) were machined to form lips around the main cylinder, into which were inserted solid platinum electrodes (10 mm diameter, 1 mm thick disc, from SurePure Chemetals, USA). This gave a geometric electrode surface area of 35 mm^2^ and an inter-electrode spacing of 7.4 mm. Different types of carbon electrode materials were then cut into *ca.* 10 mm circles by hand, and inserted between the platinum electrode and the cylinder, into which the electrolyte was injected. The carbon electrodes were Pyrolytic Graphite Thermal Interface Material (0.017 mm thick, 1750 W m^−1^ thermal conductivity, RS Components, UK) or two thicknesses of flexible graphite ‘graphoil’ gasket sheets (0.3 mm thick or 1 mm thick, both from Xiaochengshop, China).

The platinum electrodes were temperature controlled by contacting with RS-TX150 thermostatic circulator baths (Grant Instruments Ltd, UK) *via* copper heat exchangers, as previously described.^4^

### Thermogalvanic characterisation methodology

All thermogalvanic measurements were allowed to reach steady state (unless otherwise specified in the manuscript), and characterisation was performed using the ‘sequence of constant voltages’ method previously reported, using either 2-point or 5-point power curve measurements.^15^ Specifically, these measurements yielded the voltage and current density (*V*_ocp_ and *j*_sc_) as well as maximum power density (*P*_max_, in W m^−2^). This was converted into the absolute maximum power generated by the cell (*p*_max_, in W) by dividing by the exposed electrode surface area (0.000094 m^2^ for the filled electrolyte cells; surface area for the three-sided gel cells are specified later). A Keysight B2901A Source Measure Unit and Quick IV software (Keysight, UK) were used throughout. For ungelled systems, current and voltage were measured for 300 s, and the average of the data from 151–300 s reported; for the gel systems, this expanded to 600 s with the average taken from 301–600 s.

### Thermogalvanic assemblies incorporating the heat flux sensor

**Fig. 2:**
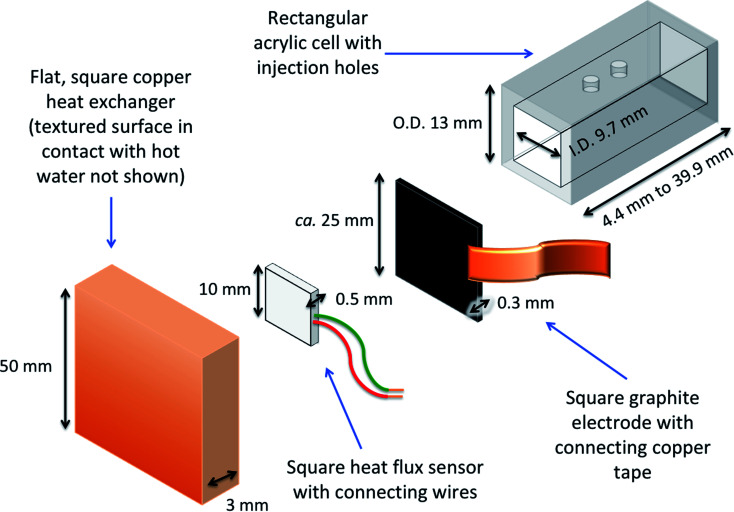
Exploded diagram demonstrating the construction of the hot electrode half of the thermogalvanic cell; the cold electrode side (not shown) comprised of another graphite electrode and a colder copper heat exchanger.

A dedicated thermogalvanic cell was developed to interface reproducibly with a commercial heat flux sensor (gSKIN-XP26 9C calibrated, greenTEC, Switzerland), which was a 10 mm by 10 mm square. The resulting assembly is described visually in Fig. 2 (a photograph is also shown later, in Fig. 4). The thermostatic water baths, copper heat exchangers and measurement techniques were unchanged from above, but the main cell was a hollow rectangular tube (extruded, clear acrylic hollow square tubing, 13 mm diameter and 9.7 mm bore, eBay Co. UK), which was cut to shape to vary the electrode separation distance, and had two injection holes drilled in the top. Squares of the 0.3 mm thick graphite were cut out and held in place at both ends of the cell by compressing it between the cell and the heat exchangers; the heat flux sensor was also compressed between the graphite electrode and hot copper heat exchanger. The electrodes and heat exchanger were both larger than the heat flux sensor, ensuring that all conductive thermal energy passing from the hot side into the thermogalvanic cell had to pass through the heat flux sensor (an irradiation effect could occur through the air gaps, but this has been modelled elsewhere^22^ and is expected to be negligible). Repeated use of the graphite resulted in increasing deformation and therefore worsening thermal contact, hence the electrodes could not be glued and each electrode was only used for one experiment before being disposed of. This arrangement gave an electrolyte-exposed square electrode surface area, *A*, of 94 mm^2^.

Photographs and *IR* images were obtained using an iPhone 11 Pro Max (Apple Inc., USA) and a Seek Thermal CompactXR with iPhone connector (Seek Thermal Inc., USA), respectively.

### Gelled electrolyte preparation and cell

Electrolytes were partially or fully gelled by the addition of 1.5 wt% or 3 wt% equivalents of sodium polyacrylate powder (SnoWonder Instant Snow Mix, SnoWonder, USA), which achieved the majority of its swelling within *ca.* 1 minute. Physically moving the gelled electrolyte into the thermogalvanic cell was found to result in significant reproducibility issues, due to different packing, trapped air, *etc.* Reproducible measurement was only achieved by physically removing one side of the rectangular cell housing, changing it from an enclosed square into a U-shape. All measurements reported here were recorded in three cells with electrode separation of 13.6, 20.9 or 29.2 mm; these cells were set up and the heat flux through the empty three-sided cell was measured. Then 0.4 M K_3_/K_4_[Fe(CN)_6_] electrolyte was added to fill the cell by *ca.* 80%; for 13.6, 20.9 or 29.2 mm this was 1, 1.57 and 2.15 mL, respectively, affording electrode–electrolyte surface areas of *ca.* 71, 71 and 73 mm^2^, respectively. The electrolyte was allowed to thermally equilibrate, measured, and then either 1.5 wt% or 3 wt% equivalents of sodium polyacrylate powder was added through the open side of the cell. This resulted in a homogenous dispersion of the powder and reproducibly formed a fully packed, air bubble-free gel. The system was measured using the thermogalvanic characterisation methodology described above, when the output from the heat flux sensor reached steady state (*ca.* 5 min).

### Conversion of the heat flux sensor output into heat flux

**Fig. 3:**
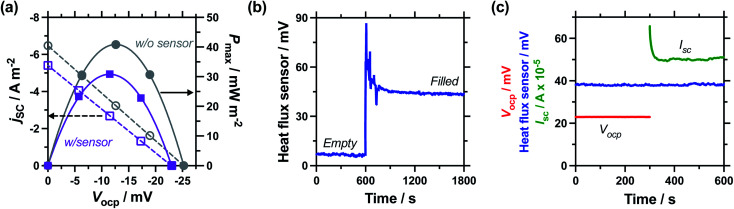
Figure showing (a) power curves of the 0.4 M K_3_/K_4_[Fe(CN)_6_] thermocell both with (purple) and without (grey) the heat flux sensor thermally in-series. Also (b) the voltage measured with time when exposed to Δ*T* = 20 K; first recording the empty thermocell for 600 s, then the cell was filled with 0.4 M K_3_/K_4_[Fe(CN)_6_]. Also shown (c) is raw data recorded for a 2-point thermogalvanic comparison of a thermally equilibrated cell, showing the open circuit potential (*V*_ocp_, red) and then the short-circuit current (*I*_sc_, green); the heat flux sensor output (blue) as also measured throughout. All data recorded at Δ*T* = 20 K with an electrode separation of 29.3 mm.

The potential difference generated across the commercial heat flux sensor (gSKIN-XP26 9C calibrated, greenTEC, Switzerland) was recorded every 0.25 s using chronopotentiometric measurements *via* a potentiostat (PGSTAT204 potentiostat with NOVA 2 software, Metrohm, UK). It was found that disabling the auto-ranging on the current and selecting the smallest current option (*i.e.* highest impedance) improved the reliability of the measurements as it suppressed any parasitic thermoelectric processes. Raw output data is presented later in Fig. 3 and 6.

**Fig. 4:**
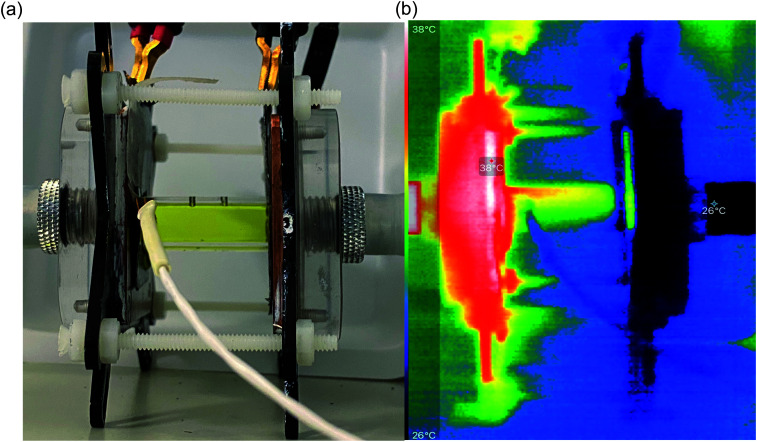
Photos of (a) the actual thermogalvanic cell, sandwiched between the two heat exchangers (the white wire coming from the left is the heat flux sensor wiring); electrode separation = 29.3 mm, Δ*T*_app_ = 20 K; and (b) *IR* image of the same cell, going from white = hottest to black = coldest.

The heat flux sensor is a thermoelectric module designed for ultra-high resolution of conductive heat flux, and the potential difference generated across the sensor (Δ*V*_s_) was converted into heat flux using the manufacturer-supplied sensitivity factor of 15.09 μV per W m^−2^ of heat flux (at 295.65 K).^38^ The temperature-corrected sensitivity factor for this particular sensor was determined using the supplied 15.09 + 0.0189 (*T*_s_ − 295.65) relationship, where *T*_s_ is the temperature of the sensor in K. This value for *T*_s_ can be approximated using the temperature of the hot heat exchanger, *T*_h_, except the heat flux sensor acted as an additional thermal resistance in-series between the heat exchanger and graphite electrode meaning it was not as hot as the copper. Therefore, *T*_s_ was instead calculated for each experiment by first calculating the experienced temperature difference across the cell using:

7 Δ*T*_exp_ = *V*_ocp_/*S*_e_

where Δ*T*_exp_ is the experienced temperature difference across the two graphite electrodes, *V*_ocp_ is the measured potential difference (in mV), and *S*_e_ is the thermogalvanic Seebeck coefficient of the electrolyte (−1.4 mV K^−1^). This will be equal to or smaller than the applied temperature difference (Δ*T*_app_), given by *T*_h_ − *T*_c_, where *T*_h_ represents the temperature of the hot heat exchanger and *T*_c_ the temperature of the cold heat exchanger. Assuming the majority of the difference between Δ*T*_app_ and Δ*T*_exp_ is lost over the heat flux sensor, then *T*_s_ is given by

8 *T*_s_ = *T*_h_ − (Δ*T*_app_ − Δ*T*_exp_)

Most experiments used *T*_h_ = 313.15 K. While the *T*_s_ varied as a function of cell and experiment, it fell within the narrow range of 308.45 to 309.85 K; the values for Δ*T*_exp_ are included in Table S2.† Arguably an even more accurate value would have been the value halfway between *T*_h_ and *T*_s_ (*i.e.* 0.5(*T*_h_ + *T*_s_)) given that these values represent the temperature gradient across the sensor, but this additional correction factor was found to have only a very minor effect upon the heat flux values.

As such, the measured total heat flux (*q*_total_, in W) through the cell is given by:

9 *q*_total_ = *A*_s_(15.09Δ*V*_s_ + 0.0189)((*T*_h_ − (Δ*T*_app_ − *V*_ocp_/*S*_e_)) − 295.65)

where *A*_s_ is the known surface area of the heat flux sensor (0.0001 m^2^), *S*_e_, *T*_h_ and Δ*T*_app_ are known, while Δ*V*_s_ and *V*_ocp_ were the simultaneously measured outputs from the heat flux sensor and the thermogalvanic cell, respectively. This value in W could be converted into the total heat flux density (*Q*_total_, in W m^−2^) by omitting *A*_s_ from this calculation.

This procedure was performed first using the cell assembly without electrolyte, and then the cell was filled *in situ* and the measurement repeated. The heat flux for the empty cell, *q*_empty_, was subtracted from the filled cell value, *q*_total_, to afford the electrolyte-only, measured heat flux value, *q*_m_.

### Calculation of the estimated heat flux

The estimated heat flux was calculated using the often-reported adaption of Fourier's Law (eqn (6)) except the estimated absolute heat flux (*q*_e_, in W) was explicitly calculated from experimental data using:10
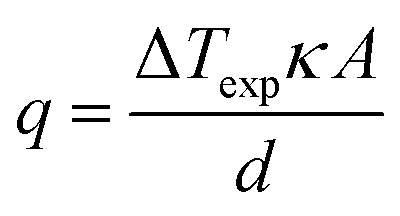
where Δ*T*_exp_ was experimentally determined as described above, *κ* was the reported^34,37^ thermal conductivity of similar concentrations of aqueous potassium ferri/ferrocyanide of 0.55 W m^−1^ K^−1^, *A* was the cross-sectional area of the electrode–electrolyte interface at the hot electrode (0.000094 m^2^), and *d* was the distance between the two electrodes (ranged between 0.0044 and 0.0399 m). The estimated heat flux density (*Q*_e_, in W m^−2^) could be calculated by omitting *A* from eqn (10).

### Calculation of the efficiency values

The absolute maximum power of the thermogalvanic cell (*p*_max_) was measured; the absolute efficiency of the electrolyte's thermogalvanic performance, *η*, was then determined using both the experimentally measured heat flux (*q*_m_) and the heat flux estimated using Fourier's Law (*q*_e_). The absolute efficiency of conversion was given by the ratio of *p*_max_ to *q* (*cf.*eqn (4)).

Efficiency relative to the theoretical Carnot cycle maximum, *η*_r_, was determined using the fractional relationship in the Introduction (*cf.*eqn (5)) except instead of using the applied *T*_h_ value, the temperature of the heat flux sensor, *T*_s_, was used. The *T*_s_ was determined based upon the measured *V*_ocp_, as described by eqn (7) and (8).

### Empty cell heat flux and the total device efficiency

As noted above, the measured total heat flux, *q*_total_, was corrected to measure the electrolyte-only value, *q*_m_, by subtracting the heat flux of the empty cell, *q*_empty_; these measured values of *q*_empty_ are tabulated in the ESI.† It is also possible to predict the heat flux through the acrylic cell using the eqn (10), using the *κ* value for this plastic (*ca.* 0.20 W m^−1^ K^−1^) and the surface area of the plastic (0.000075 m^2^) that made up the face of the hollow square. Typically this predicted value was *ca.* 40% of the total measured empty cell heat flux, as shown later in this paper. As for the remaining measured heat flux for the empty cell, it is estimated that the majority of this was lost *via* conduction through the graphite electrode and into the metallic crocodile clip and wiring at the hot electrode side. The remainder would have been lost to the surroundings and air *via* radiation and air convection, respectively (from the plastic cell body, graphite electrode, metallic clip and wiring).

As these factors were neither systematically investigated nor optimised, the majority of this paper focuses upon electrolyte-only efficiency parameters. However, the measured total device efficiency parameters can be derived by eqn (4) and (5), but using *q*_total_ instead of *q*_m_; all measured values are tabulated in Tables S2 and S3.†

## Results and discussion

### Initial selection of the electrode material

As summarised in the Introduction, to the best of our knowledge the direct heat flux through thermogalvanic cells has not been quantified. This was achieved here by putting a heat flux sensor thermally in-series with the cell at the ‘hot’ electrode, as shown in Fig. 1(d). This, in conjunction to the quantification of the absolute thermogalvanic power, allowed unambiguous comparison of the ratio of the two.

A detailed explanation of this set-up is included in the Experimental section. However, a flexible electrode material was required to work reproducibly in this set-up, and therefore a range of electrode materials were screened using a conventional thermogalvanic cell setup. This was achieved using a previously-reported^4^ thermocell setup, using solid platinum electrodes, filled with 0.2 M K_3_[FeCN_6_] and 0.2 M K_4_[FeCN_6_] (or 0.4 M K_3_/K_4_[Fe(CN)_6_]), exposed to a temperature gradient of 20 K, and measured using the formalised sequence of potentials.^15^ Three different types of graphite materials were then evaluated by placing them between the platinum and the electrolyte.

The resulting thermogalvanic steady-state outputs are summarised in Table 1; clearly platinum possessed the optimum performance due to the high output current density expected of such a highly electrocatalytic electrode towards [Fe(CN)_6_]^3−/4−^ electron transfer kinetics,^4^ but this electrode material was too firm to reproducibly interface with the heat flux sensor and could not be utilised further. Pyrolytic crystalline graphite was bought as a thermal interface material, and it displayed excellent thermal properties (actually increasing the *V*_ocp_ when placed on top of the Pt), but also demonstrated very poor electrocatalytic properties with the current decreasing *ca.* 60-fold. Two thicknesses of amorphous gasket (graphoil) graphite were also evaluated; as the thickness increased the thermal resistance also increased, resulting in a drop in *V*_ocp_. However, the current was far higher than that recorded at the crystalline graphite and increased as the thickness increased; this is likely due to partial porosity of the graphoil material increasing the electrochemically active surface area, and exposed electrocatalytic edge sites^39^ that comes with its expansion and compression during manufacturing.^4^ The 0.3 mm thick graphite was chosen as it was suitably thin and deformable while maintaining reasonable electrocatalytic ability, although it's important to note it generates only *ca.* 35% of the power of pure platinum (40 mW m^−2^*vs.* 114 mW m^−2^).

**Table 1:** Comparison of the thermogalvanic performance as a function of electrode material, when exposed to Δ*T* = 20 K

Electrode material	Electrode thickness/mm	−*V*_ocp_/mV	−*j*_sc_/A m^−2^	*P*_max_/mW m^−2^
Platinum	N/A	24.6	18.5	114
Pyrolytic graphite	0.017	24.8	0.3	2
Amorphous graphite	0.3	24.3	6.6	40
Amorphous graphite	1.0	23.5	11.9	70

### Introduction of the heat flux sensor thermally in-series

Next, the effect of introducing the heat flux sensor was explored, using the novel rectangular cell design described in detail in the Experimental section. Firstly, the thermogalvanic power was measured. Fig. 3(a) displays overlaid power curves for a rectangular cell with an electrode separation of 29.3 mm, filled with 0.4 M K_3_/K_4_[FeCN_6_] electrolyte, and equilibrated at an applied Δ*T* = 20.0 K (*T*_c_ = 20.0 °C, *T*_h_ = 40.0 °C). Power curves were recorded for the same cell with (grey circles) and without (purple squares) the heat flux sensor thermally in-series. The heat flux sensor clearly added additional thermal resistance, reducing the *V*_ocp_ and therefore the current and the power; despite this, an ideal power curve was still obtained for the thermogalvanic cell power output. Using a thermogalvanic *S*_e_ value of −1.4 mV K^−1^,^4,5,11,40,41^ for the electrolyte, it implies an applied Δ*T*_app_ = 20.0 K translated into the graphite electrodes experiencing Δ*T*_exp_ = 16.4 K with the heat flux sensor in-series.

Next, the output from the heat flux sensor was quantified. Fig. 3(b) displays the voltage output from the heat flux sensor when connected thermally in-series with an empty cell (same cell and conditions as above). A constant output of *ca.* 7 mV was recorded, corresponding to a heat flux of *ca.* 45 mW (or heat flux density of 450 W m^−2^) through the sensor. After 10 min the 0.4 M K_3_/K_4_[FeCN_6_] electrolyte was injected to fill the cell, and a sharp spike in voltage (indicating increased heat flux) was observed; this dropped over *ca.* 5 min as the solution came to temperature, with further spikes and dips in this period due to injecting and extracting liquid from the cell in order to remove all the trapped bubbles. This equilibrated to give a constant value of *ca.* 44 mV, or a total measured heat flux of *q*_total_ = 295 mW; this was how the heat flux for a filled cell and the corresponding empty cell were measured, and unless specified otherwise the empty cell-corrected electrolyte heat flux values were used, *e.g. q*_m_ = 295 mW − 45 mW = 250 mW.

Power was characterised for all subsequent cells using 2-point measurements, *e.g.* measuring just *V*_ocp_ and *j*_sc_, and determining the power density using *P*_max_ = 0.25*V*_ocp_*j*_sc_.^15^Fig. 3(c) displays a 10 min characterisation measurement after the cell has come to thermal equilibrium; the *V*_ocp_ for the first 300 s is shown and was very stable, followed by *I*_sc_ measurement (then converted from *I*_sc_ to *j*_sc_ by dividing by the electrode surface area). An initial drop in current is observed as concentration gradients are established at the two electrodes, but it rapidly comes to equilibrium as the rate of consumption and mass transport equilibrate, resulting in genuine steady state power generation.^15^ The average from 151–300 s was used to quantify both *V*_ocp_ and *j*_sc_. The simultaneous measurement of heat flux through the sensor was also measured (as overlaid in Fig. 3(c)), and this didn't display any significant changes, even when current was allowed to flow through the thermogalvanic cell.

The cell was found to generate *ca.* 31 ± 3 μW thermogalvanic power at steady state (from triplicate measurements), whereas the cell-corrected electrolyte heat flux value was *ca.* 236 ± 17 mW; this equates to an absolute efficiency of 0.013 ± 0.002%, or 0.024 ± 0.003% *vs.* Carnot efficiency. Conversely, the estimated heat flux using the typical model employed for thermogalvanic cells of 1D transport through a solid (eqn (6)) predicted nearly an order of magnitude less heat flux at 30 ± 1 mW. This results in an estimated Carnot efficiency of 0.19 ± 0.02%, *i.e.* the estimated efficiency was nearly 8-fold higher than the directly measured efficiency of conversion. This value increases to 9-fold if the total heat flux (*q*_total_) of the entire device is used, rather than the empty cell-corrected electrolyte heat flux value (*q*_m_).

In order to identify the source of this additional heat flux, *IR* imaging was employed. Fig. 4 compares photos *vs. IR* images of the setup, and the latter clearly indicates how the top of the cell was significantly hotter than the bottom, indicating a significant amount of convection is occurring; this in turn results in significant radiation heat loss from the top of the cell. Neither convective nor radiation heat-transfer mechanisms are considered by the 1D Fourier's Law prediction (eqn (6)), thus accounting for the very significant difference observed here between actual measurement of the heat flux *vs.* the routinely employed estimated heat flux.

The implications of this order-of-magnitude difference, and a comparison against other published values are both discussed in detail at the end of this paper. Given the observed key role of the cells external surface, different electrode separations were evaluated next.

### Effect of the inter-electrode separation distance

**Fig. 5:**
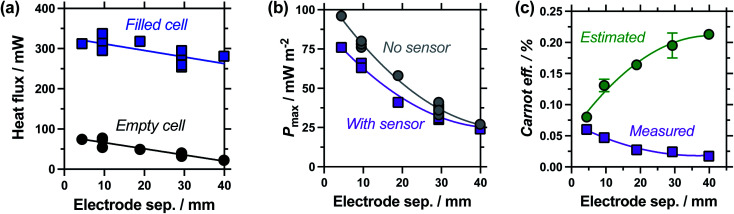
Study of the effect of electrode separation distance, showing (a) plot of the heat flux through the empty and electrolyte-filled cells; (b) the thermogalvanic power density with and without the heat flux sensor in-series; and (c) the estimated and measured Carnot-relative efficiencies of thermogalvanic conversion. All measured at Δ*T*_app_ = 20 K; all repeat measurements for 9.5 mm and 29.3 mm are shown in (a) and (b), with the combined errors shown in (c) as the 70% confidence interval (1 standard deviation), if larger than the symbol.

It has been previously reported that the power density of a thermogalvanic cell drops significantly with increasing inter-electrode separation, but power conversion efficiency will increase.^27,40,42^ Given this expected relationship, we set out to measure efficiency over 5 different electrode separations, all at an applied Δ*T* = 20 K. The results are summarised in Table 2 (full experimental results for cells without the heat flux sensor are in Table S1,† and with the heat flux sensor in Table S2†); key results are visualised in Fig. 5.

**Table 2:** Summary of the thermogalvanic power and heat flux measurements as a function of electrode separation distance (*d*, top) and applied temperature difference (Δ*T*, bottom) to afford measured efficiencies (absolute, *η*_m_, and relative to the Carnot cycle, *η*_r,m_). These are compared against the estimated heat flux (*q*_e_) and associated estimated efficiencies. Error values indicated by (±) are the standard deviation of between 3 to 5 repeat measurements. See Table S1 for equivalent studies without more heat flux in-series, and Table S2 for a more comprehensive version of this table

Experimental conditions			Measurement results		Measured efficiencies		Estimated heat flux and estimated efficiencies		
*d*/mm	*T*_h_/°C	*T*_c_/°C	*p*_max_ */*μW	*q*_m_/mW	*η*_m_/10^−3^ %	*η*_r,m_/%	*q*_e_/mW	*η*_e_/10^−3^ %	*η*_r,e_/%
4.4	40	20	7.1	239	3.0	0.060	180	4.0	0.08
9.5	40	20	6.1 (±0.1)	248 (±24)	2.4 (±0.2)	0.047 (±0.005)	87.5 (±3.6)	6.9 (±0.3)	0.13 (±0.01)
18.9	40	20	3.9	268	1.4	0.027	44.8	8.7	0.16
29.3	40	20	3.1 (±0.3)	236 (±17)	1.3 (±0.2)	0.024 (±0.003)	29.6 (±1.2)	10.4 (±0.1)	0.19 (±0.02)
39.9	40	20	2.3	259	0.9	0.017	20.7	11.0	0.21
9.5	40	20	6.1	248	2.4	0.047	87.5	6.9	0.13
9.5	45	20	9.5	295	3.2	0.051	107	8.9	0.14
9.5	50	20	13.8	368	3.7	0.050	128	10.8	0.15
9.5	55	20	18.6	439	4.2	0.050	149	12.5	0.15
9.5	60	20	26.4	584	4.5	0.048	164	16.1	0.17

**Fig. 6:**
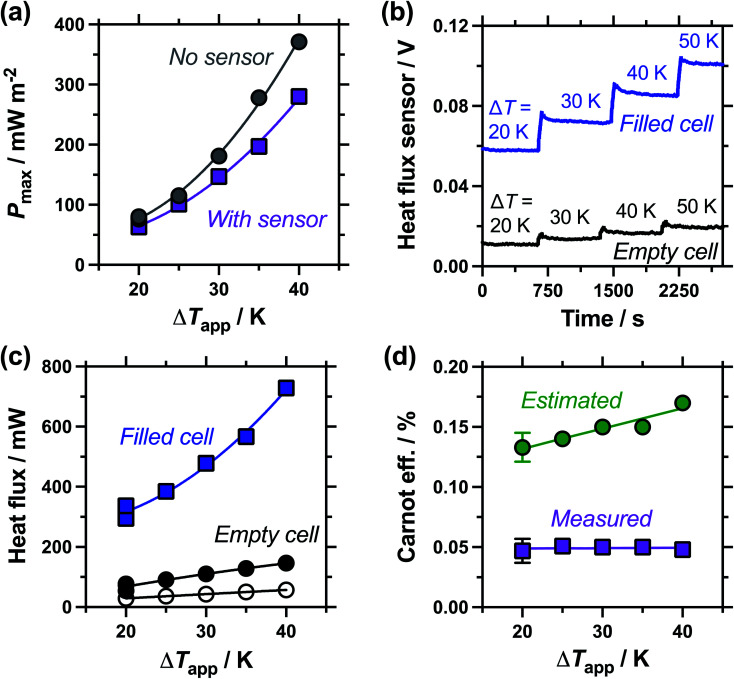
(a) Plot of the maximum thermogalvanic power produced as a function of applied temperature difference, with (purple square) and without (grey circles) the heat flux sensor thermally in-series; (b) the raw heat flux sensor output as a function of applied temperature difference, with the thermogalvanic cell empty and filled with electrolyte, and (c) the corresponding heat flux derived from the raw data for the electrolyte-filled (square) and empty (filled circle) cell; also shown is the estimated heat flux expected by conduction through the perspex cell (empty circles); (d) the Carnot relative efficiency of thermogalvanic conversion using the estimated (green circles) and measured (purple squares) heat flux values, using electrolyte-only values (*i.e.* filled cell minus empty cell, for the measured heat flux). All measured using an electrode separation of 9.5 mm. All triplicate measurements for Δ*T*_app_ = 20 K are shown in (a) and (c), with the error bars in (d) corresponding to the combined errors as the 95% confidence interval (2 standard deviations).

Measuring the heat flux through the empty and filled cells (Fig. 5(a)), the heat flux decreased in a linear manner as the electrode separation of the empty cells increases, in line with expectations for frustrated conduction through the longer plastic. A similar trend was observed in the electrolyte-filled cells, meaning that the corrected heat flux was essentially constant for all the cells, regardless of the electrode separation. This trend of largely constant heat flux *vs.* electrode separation deviates from the calculated heat flux, which considers conduction only and predicts the overall heat flux should decrease with increasing separation. It is likely that as the cell becomes longer, conduction decreases whereas convection and radiation increases, with these two effects thus cancelling each other out. As the electrode separation increases power also decreases exponentially (plotted in Fig. 5(b)), in line with expectations for a mass-transport limited thermogalvanic cell and prior studies.^27,40^ This trend was observed both with and without the heat flux sensor in series.

Fig. 5(c) compares the Carnot efficiency for the cells as a function of electrode separation, using either the genuinely measured heat flux or estimated heat flux; the absolute efficiency values followed the same trend (Fig. S1(a)†). Interestingly, the smallest cell shows fair correlation between measured and estimated efficiencies, consistent with the smallest cell being a conduction-dominated system. Our estimated heat flux model predicts increasing efficiency with increasing electrode separation, but with increasingly diminishing returns; this is in excellent agreement with earlier fundamental work.^27^ However, the genuinely measured heat flux results indicate decreasing efficiency, due to frustrated ion transport yet convection-boosted heat loss. This complete divergence of prior predictions *vs.* genuine measurements is of significance for future cell design and optimisation.

### Effect of the applied temperature difference

Following the investigation into inter-electrode separation, the effect of temperature difference was explored, using a fixed, 9.5 mm electrode separation. Here the temperature difference was increased from Δ*T*_app_ = 20 K to Δ*T*_app_ = 40 K, by increasing the temperature of the hot electrode. As shown in Fig. 6(a), the power increased as the Δ*T*_app_ increased, in line with expectations.^5,15^ While the trends in *P*_max_ with and without the heat flux sensor in-series deviated *vs.* Δ*T*_app_, the two trends were comparable if plotted *vs.* Δ*T*_exp_ (as shown in Fig. S1(b)),† as this corrects for the additional thermal resistance introduced by the sensor.

Fig. 6(b) displays the raw data output by the heat flux sensor in-series with both the empty cell and the electrolyte-filled cells, whereas Fig. 6(c) plots the actual heat flux as a function of Δ*T*_app_ (the same trend exists *vs.* Δ*T*_exp_). The heat flux measured for the empty cell increased in a linear manner in line with Fourier's Law, whereas the electrolyte-filled cell increased in a non-linear manner, consistent with enhanced convection at greater values of Δ*T*. Also overlaid in Fig. 6(c) is the predicted heat flux calculated solely for conduction through the perspex cell using Fourier's Law (hollow circles), which is only *ca.* 40% of the heat flux measured for the whole device. Therefore some additional thermal energy is presumably lost to radiation and air-convection, with the majority lost to conduction through the electrode and into the wiring; this parasitic thermal lost is well recognised,^43^ and device-design requires two dissimilar thermogalvanic cell chemistries to be employed to help combat this thermal short-circuit.^18,43^ This effect is also clearly seen in Fig. 4(b), with the image achieved *via IR* radiation heat loss, and the clip at the hot electrode also being visually warmer than the background.

Both *P*_max_ and heat flux increased with increasing Δ*T*, but *P*_max_ increased by a greater magnitude and thus the overall absolute (electrolyte-only) efficiency increased with increasing Δ*T* (shown in Fig. S1(c)†). However, this overall efficiency gain was equivalent to expected gains from a Carnot engine at the increased Δ*T*, meaning the overall Carnot relative efficiency was independent of Δ*T* (Fig. 6(d)). Once again, the estimated heat flux lacked this nuance, with both the estimated absolute efficiency and estimated Carnot efficiency values increasing with Δ*T*. The divergence in these trends, combined with the smaller estimated heat flux, resulted in the estimated *vs.* measured efficiency values differing by a factor of 4.5 by Δ*T*_app_ = 40 K, in this 9.5 mm separation cell.

### Effect of gelling the electrolyte

Gelled or ‘*quasi*-solid’ electrolytes have appeared numerous times, *e.g.*^9,10,12–14,44–48^ in thermogalvanic cells as a method of producing an electrolyte which is not susceptible to leaking and so supported the development of wearable devices;^9,10^ theoretically it also reduces the thermal conductivity through the thermocell. We therefore investigated the effect of gelling the electrolyte, especially since gelation was expected to ‘switch off’ the convection found to be so influential earlier in this study. This was performed by adding either 1.5 wt% or 3.0 wt%-equivalent of sodium polyacrylate powder; this is a textured, superabsorbent material that can rapidly swell and even gel highly concentrated electrolytes, within seconds.^22^

**Fig. 7:**
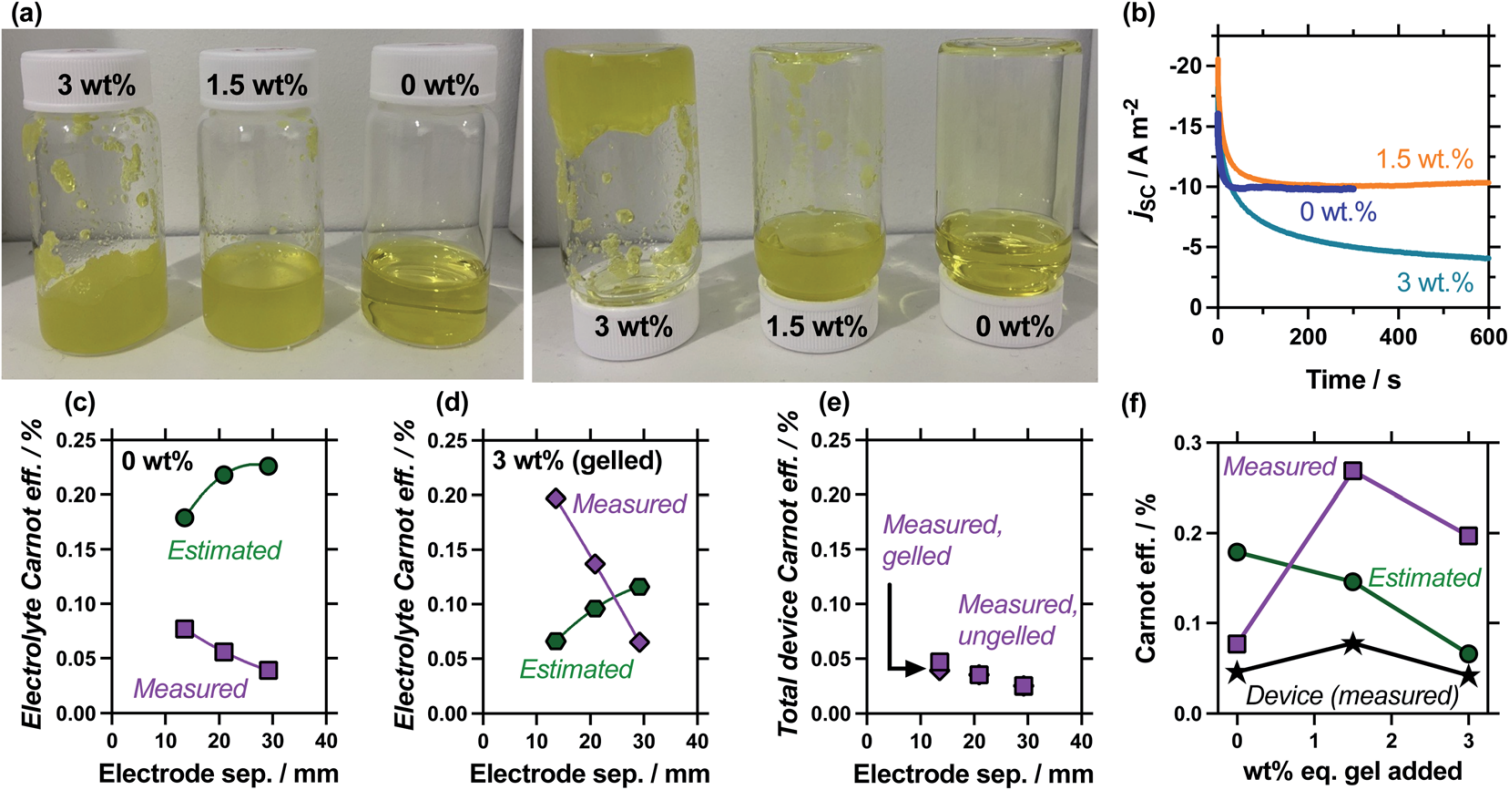
Showing (a) photos of the as-prepared 0.4 M K_3_/K_4_[Fe(CN)_6_] solutions containing 0, 1.5 or 3 wt% eq. of sodium acrylate powder (left) before and (right) 60 seconds after being inverted, demonstrating only the 3 wt% eq. system was sufficiently gelled to pass the inversion test. Also (b) the measured *j*_sc_*vs.* time for these systems, showing how both 0 wt% and 1.5 wt% eq. resulted in steady-state current (half the 0 wt% data excluded for clarity) while 3 wt% failed to reach steady state over 10 min (electrode separation of 13.6 mm). Also shown is an electrode separation study, with the estimated and measured electrolyte Carnot efficiencies for (c) 0 wt% and (d) 3 wt% eq. sodium acrylate powder systems. Shown in (e) is the total device Carnot efficiency, showing largely equivalent values for the ungelled (0 wt%) and gelled (3 wt%) systems. Finally (f) plots the various Carnot efficiency values for different wt% values of sodium acrylate powder in the 13.6 mm cell. All calculations in (c–f) used the average from 2 to 4 repeat measurements, with the *j*_sc_ values averaged from 301 to 600 s; all measured at Δ*T*_app_ = 20 K in a partially filled 3-sided cell (see Experimental for full details).

Fig. 7(a) shows photographs of these systems being exposed to the inversion test, which demonstrates that 0.4 M K_3_/K_4_[FeCN_6_] electrolyte containing 1.5 wt% eq. sodium polyacrylate powder forms a heterogeneous, free-flowing slurry, whereas 3.0 wt% eq. results in a genuinely gelled electrolyte. Table S3† summarises all relevant values, while Fig. 7(b) plots the measurement of the *j*_sc_*versus* time; addition of 1.5 wt% equivalent gelling agent resulted in a slightly slower equilibration time before steady state current was achieved, but otherwise didn't change the *j*_sc_, whereas the gelled 3.0 wt% equivalent system failed to reach equilibrium. The latter observation is common with fully-gelled electrolytes, which frustrate the transport of ions to such an extent that concentration imbalances accumulate and persist at the electrode surfaces.^14^

The comparison of gelled *vs.* ungelled electrolytes was also explored as a function of electrode separation. Fig. S2† plots the measured thermogalvanic powers, both with and without the heat flux sensor in-series, while Fig. 7(c) and (d) summarise the electrolyte-only Carnot efficiency values. The ungelled system (0 wt%, Fig. 7(c)) displayed the expected deviation between estimated and measured values (*cf.*Fig. 5(c)), but significant differences were observed in the gelled system (3 wt%, Fig. 7(d)). In the gelled system, measured efficiency still decreased with electrode separation while estimated efficiency increased. However, the estimated efficiency values were significantly lower, because the power generated by the gelled systems was lower but the predicted heat flux remained unchanged (as *κ* = 0.55 W m^−1^ K^−1^ was assumed throughout). Conversely, the measured actual heat flux was significantly reduced upon gelation; because heat flux was reduced even more than the current was reduced, the measured efficiency actually increased significantly. This difference means at relatively small electrode separations (<20 mm), the measured gelled electrolyte efficiency actually exceed estimated efficiency.

These results highlight how the assumption of *κ* = 0.55 W m^−1^ K^−1^ is flawed for both convective (*e.g.* liquid) and convection-supressed (*e.g*. gelled) systems. This mirrors the observations of Yu *et al.* who estimated the effective thermal conductivity, *κ*_eff_, of their [Fe(CN)_6_]^3−/4−^ electrolyte using *IR* imaging; while the conduction-only thermal conductivity should have been *ca.* 0.55 W m^−1^ K^−1^,^34,37^ at an applied Δ*T* = 50 K the effective thermal conductivity was 1.64 W m^−1^ K^−1^; after guanidinium-induced crystallisation this dropped down to *ca.* 0.4 W m^−1^ K^−1^,^23^ with the crystals presumably physically causing a reduction in heat flux. Additionally, Pu *et al.* reported *κ* values in the range of 0.31 to 0.38 W m^−1^ K^−1^ for a crosslinked polyacrylamide hydrogel monolith saturated with a 0.1 M K_3_/K_4_[Fe(CN)_6_] + 5.4 M LiBr electrolyte.^48^ Different *κ*_eff_ values were applied to our model such that the estimated electrolyte-only heat flux matched the measured heat flux; this gave *κ*_eff_ = 1.28 W m^−1^ K^−1^ for the 0 wt% system in the 13.6 mm cell, and *κ*_eff_ = 0.18 W m^−1^ K^−1^ for the 3 wt% system. However, *κ*_eff_ is also a cell-dependant value, and increased in the 20.9 mm cell to 2.14 W m^−1^ K^−1^ and 0.39 W m^−1^ K^−1^ for the gelled and ungelled systems, respectively, likely due to increased convective and radiative losses as the cell gets larger.

An additional concept that has apparently never been examined before is total device efficiency. Throughout, the electrolyte-only heat flux has been employed, but total heat flux was quantified. The measured Carnot efficiency was recalculated using the total heat flux, and these values are plotted in Fig. 7(e). It demonstrates that device-efficiency was essentially indistinguishable for gelled and ungelled electrolytes. This was because even though heat flux was suppressed more than current flow upon gelation, the additional heat flux through the cell assembly itself became a much more significant fraction of total heat flux, and reduced the overall total device efficiency. More thermally resistive electrolytes therefore require more thermally resistive cell housings in order to achieve boosts in efficiency in genuine ‘real world’ whole device applications.

Finally, the 1.5 wt% eq. gelling agent yielded a heterogeneous suspension; the ionic, solid particles appear to frustrate bulk thermal transfer (such as convection, and even to a degree conduction) but remains a highly ionically conductive system, boosting the (genuine) efficiency relative to the electrolyte alone. The measured and estimated Carnot relative efficiency is plotted in Fig. 7(f) for 0 wt%, 1.5 wt% and 3 wt% eq. sodium polyacrylate in the 13.6 mm cell. These values demonstrate the heterogeneous suspension caused by 1.5 wt% eq. possesses optimal conditions and displays the highest efficiency; this was reflected in an additionally boosted total device efficiency. Broadly, these precise measurements prove an often stated but only previously tentatively proven^22^ concept; that gelled and *pseudo*-gelled electrolytes can result in genuinely more efficient conversion in thermogalvanic cells, provided they selectively frustrate heat flux more than current generation.

### A comparison of the factors and values *vs.* previous reports

Table 3 summarises a number of published papers that quote estimated Carnot efficiency values (using directly measured thermogalvanic power and estimated heat flux for the electrolyte only). The table is not an exhaustive summary of the literature, and is restricted to studies using aqueous [Fe(CN)_6_]^3−/4−^; it primarily summarises fundamental studies employing planar platinum electrodes (by Quickenden *et al.*^24,25,27^ or Lee *et al.*^37^) who performed limited temperature, orientation and concentration studies, as well as three high surface area carbon electrode studies as exemplars,^23,34,40^ and stainless steel as a comparison.^22^ A number of other studies that use nanostructured electrodes have been excluded, because that is an additional factor not studied here; others have reported seemingly promising systems, *e.g.* [Fe(CN)_6_]^3−/4−^/KCl gelatine hydrogels with a *S*_e_ up to 17 mV K^−1^,^12^ but didn't report sufficient thermogalvanic characterisation parameters such that their performance can be compared with others.

**Table 3:** Comparison of estimated Carnot efficiency values from this work and a non-exhaustive range of studies where aqueous [Fe(CN)_6_]^3−/4−^ electrolyte was used, and a range of experimental conditions (such as electrode material, electrode separation, temperature difference and cell orientation were studied). All estimated efficiency using the estimated heat flux, except ref. 23 and this work^a^

Aqueous electrolyte composition	Δ*T*/K	Electrode	Electrode sep./mm (orientation)	Estimated Carnot efficiency/%	Measured Carnot efficiency/%	Ref.
0.1 M K_3_[Fe(CN)_6_] + 0.1 M K_4_[Fe(CN)_6_]	20	Stainless steel	*ca.* 2	0.0002		22
0.1 M K_3_/K_4_[Fe(CN)_6_]^b^	20	SWCNT sheet electrode	10	0.0010	—	40
			40 (parallel)	0.0028		
0.4 M K_3_/K_4_[Fe(CN)_6_]^b^	51	CNT scroll^c^	25 (parallel)		—	34
		(i)		∼1.2		
		(ii)		∼2.2		
		(iii)		∼3.6		
		(iv)		3.95		
0.4 M K_3_[Fe(CN)_6_] + 0.4 M K_4_[Fe(CN)_6_]	10	Porous carbon fabric paper on graphite	15 (c-o-h)	∼0.68^d^		23
	50			∼0.48^d^		
0.26 M K_3_[Fe(CN)_6_] + 0.26 M K_4_[Fe(CN)_6_] + 0.8 M KCl	20	Pre-treated Pt foil	30 (h-o-c)	0.44 (*t* = 16 s)	—	25
				0.11 (*t* = 600 s)		
0.26 M K_3_[Fe(CN)_6_] + 0.26 M K_4_[Fe(CN)_6_] + 0.8 M KCl	20	Pre-treated Pt foil	100 (parallel)	0.50	—	24
			(c-o-h)	0.50		
			(h-o-c)	0.17		
0.26 M K_3_[Fe(CN)_6_] + 0.26 M K_4_[Fe(CN)_6_] + 0.8 M KCl	30	Pre-treated Pt foil	3	0.08	—	27
			1500 (c-o-h)	0.60		
0.4 M K_3_/K_4_[Fe(CN)_6_]^b^	15	Pt foil	8 (parallel)	0.288	—	37
0.9 M K_3_/(NH_4_)_4_[Fe(CN)_6_]^b^	25			0.276		
	35			0.271		
	15			0.392		
	25			0.417		
	35			0.399		
All 0.2 M K_3_[Fe(CN)_6_] + 0.2 M K_4_[Fe(CN)_6_]	20	0.3 mm thick amorphous graphite	9.5	0.133	0.047	This work
+0 wt% eq.	40		9.5	0.172	0.048	
+1.5 wt% eq.	20		4.4	0.080	0.060	
+3.0 wt% eq.	20		39.9	0.213	0.017	
+3.0 wt% eq. sodium polyacrylate	20		13.6	0.179	0.077	
	20		13.6	0.146	0.269	
	20		13.6	0.066	0.197	
	20		29.2 (parallel)	0.116	0.065	

a For orientation c-o-h and h-o-c represents cold-over-hot and hot-over-cold electrode arrangements, respectively. The symbol ∼ indicates the authors had to extract estimated values directly from graphs.

b Unclear if stated concentration is [FeCN_6_]^3−^ + [FeCN_6_]^4−^ = concentration, or [FeCN_6_]^3−^ = [FeCN_6_]^4−^ = concentration.

c A forest of CNT was drawn onto a 0.3 mm tungsten wire, and wrapped around to form a *ca*. 3 to 3.5 mm diameter scroll. Results are reported for (i) CNT scroll as prepared, (ii) scroll thermally oxidised, (iii) scroll platinised, and (iv) scroll platinised and compressed.

d The heat flux was estimated using the same equation as the other studies, except an effective thermal conductivity was calculated *via IR* imaging and used, rather than a conduction-only thermal conductivity.

Also listed in Table 3 are some estimated and genuinely measured efficiencies from this work. Recalling that all measurements were made at amorphous graphite that generates *ca.* one-third of the power of platinum electrodes, the efficiency values can in theory be converted to approximate planar platinum values by multiplying by 3 (which assumes thermal conduction routes would be unaffected by this substitution, but current roughly tripled). Doing this, the range of estimated Carnot efficiency values recorded in this study at graphite (0.066–0.213%) converted to platinum-equivalent values (*ca.* 0.2–0.6%) then sit well within the range of values reported by Lee *et al.*^37^ and Quickenden *et al.*^24,25,27^ for similar electrolytes at planar platinum electrodes (0.08–0.6%).

What stands out from the tabulated results is the general assertion of previous reports that (estimated) efficiency will increase with increasing electrode separation,^27,40^ which this study mirrors. However, the directly measured efficiency displays the completely opposite trend, which is significant for future device design.

In this study, the absolute efficiency (both estimated and measured) increased with increasing Δ*T*, as did the estimated Carnot efficiency, while the measured Carnot efficiency showed a minor decease with increasing Δ*T*. Interestingly, Lee *et al.* reported estimated Carnot efficiency values were also largely independent of Δ*T*,^37^ while Zhou *et al.* used indirect *IR* imaging to more accurately estimate the Carnot efficiency and reported a slight decrease with increasing Δ*T*.^23^ The reason for these slight differences is not currently known, but it's likely the Carnot efficiency can be taken as approximately independent of the applied Δ*T* (for conventional [Fe(CN)_6_]^3−/4−^-based cells). Regardless, the overall absolute efficiency does genuinely increase quite significantly with increasing Δ*T*, albeit it to a lesser degree than that predicted by the estimated heat flux methodology.

Aspects that are missing from this study are the affect upon the directly measured efficiency of (i) the electrolyte concentration, (ii) high(er) surface area electrode materials, (iii) orientation with respect to gravity, and (iv) when cells are incorporated in multi-cell devices. With regards to concentration, earlier results have already indicated that increasing the concentration of the redox active species increases the current and thus will also increase the efficiency. For example Quickenden and Vernon stated that increasing the concentration by an order of magnitude from 0.007 M each of [Fe(CN)_6_]^3−^ and [Fe(CN)_6_]^4−^ to 0.07 M of each (both containing 3 M KCl) increased the estimated Carnot efficiency by roughly an order of magnitude.^25^ Additionally, Lee *et al.* demonstrated that moving from 0.4 M [Fe(CN)_6_]^3−/4−^ (as K^+^ salts) to 0.9 M [Fe(CN)_6_]^3−/4−^ (as K_3_[Fe(CN)_6_] and (NH_4_)_4_[Fe(CN)_6_]) resulted in a *ca.* 50% increase in the estimated Carnot efficiency.^37^ However, increasing concentration can also have diminishing returns and in fact reduce the current in both super-concentrated^41^ and ion-pairing prone systems.^37^ While we believe the measured efficiency will change in proportion to concentration (at reasonably low electrolyte concentrations), this area nevertheless requires further study for genuine confirmation, especially given the potential impact of concentration upon heat flux through the entire cell due to density and viscosity changes.

The majority of papers quoting estimated Carnot efficiency values are those reporting nano-structured electrode studies. Nano-structuring is frequently employed to boost the estimated efficiency, since it is capable of enhancing electrocatalysis and/or dramatically increasing the electroactive surface area; the latter is particularly significant since nanostructing results in 3D areas where electron transfer can occur, but heat flux is predicted to remain a 1D process (*via* the estimated heat flux equation). The three nano-carbon entries in the table demonstrates how SWCNT resulted in poorly electrocatalytic electrodes (likely equivalent to the poor results observed in this study at the pyrolytic graphite electrodes), but the estimated efficiency could be boosted *ca.* 4000-fold upon moving to an exquisitely crafted platinum/carbon nanocomposite.^34^ Significantly, the high estimated Carnot efficiency value of 3.95% in the latter likely has a significantly lower genuine efficiency, given that the utilised cell was a long, thin glass tube that would lose significant heat *via* convection then radiation (*cf.*Fig. 4). The methodology described here can now be employed to accurately quantify the efficiency, and will not only aid cell design but also be able to answer the question of whether nanostructured electrodes affect heat flux through the device; this affect could be acting as thermal baffles to reduce total heat flux, or they could act as heat-exchange catalysts and boost total heat flux. Both observations are likely possibilities, given the diverse range of different electrode materials currently being reported.

With regards to the cell orientation, all measurements were performed in this study in a thermally side-by-side or parallel arrangement (as visualised in Fig. 4). Employing one electrode physically higher than the other (hot-over-cold and cold-over-hot arrangements) can have a very significant impact due to gravity effects upon convection, and this has been reported previously for thermogalvanic cells,^20,21,24,42,49–51^ with the former arrangement resulting in stagnation and a suppression of thermogalvanic current, while the latter can result in significant gravity-driven convective transfer of thermal energy.^21,24^ The effect of this upon the estimated efficiency can be seen in Table 3. Unfortunately, when orientation experiments were attempted in our thermocell setup, persistent leakage and bubble-formation issues at the graphite prohibited accurate measurement, with even small bubbles strongly impacting both current and heat flux through the cell. As such, a more robust cell design is required before orientation studies can be performed to measure the genuine efficiency.

Finally, convection-induced radiation heat loss was significant in this study, and this could be ‘fixed’ by employing a heavily insulated cell. However, multiple thermogalvanic cells are typically combined into a single device, with cells of similar chemistry being electrically connected to boost current, while dissimilar cell chemistry is used to boost voltage.^43^ This could be *via* two different cells electrically connected,^43^ or be a single monolithic block into which several cells have been machined.^18,41^ A high power density device cannot be achieved with thick, insulated walls installed between dozens of individual cells, and instead the wall thickness is (ideally) relatively thin. For thin walls, the significant convective heat loss visualised through the upper surface of the cell in this work takes on a different aspect; it could be absent in the centre of the device, or could result in a thermally parasitic transfer process that significantly unbalances the temperature difference and heat flux across the device. Those cells around the edge of the device will demonstrate this heat-loss mechanism (subject to orientation with respect to gravity); it could act as a thermal short-circuit holding back the efficiency of the entire device, or compromise the Δ*T* in these cells thus negatively impacting the overall power produced by the device, or could even have no effect at all (beyond external cells having a lower genuine efficiency relative to cells deep inside the device). The methodology presented in this study now enables such complex questions to be interrogated, though the use of multiple heat flux sensors.

All discussions above have focussed upon electrolyte-only values. Significantly, this study has also enabled the quantification of the ‘total device efficiency’ (*via* the methodology described in the Experimental section), but the parasitic cell contributions to heat flux were routinely subtracted throughout this work. This is largely because device contributions are so variable across groups, and were never deliberately adjusted or optimised in this study. For examples like the 9.5 mm cell (*cf.*Fig. 6), the device-only heat flux was a minor part of the total heat flux; some of which was conduction through the plastic cell, and the rest primarily lost to conduction into the wiring and radiation into the surroundings. Heat loss into the wiring has been recognised before,^43^ and is relatively easily addressed; for example connecting 100 electrolyte pairs thermally in-parallel but electrically in-series^43^ will likely reduce this parasitic effect of the external wiring by 100-fold per cell. Thinner wires will also reduce this. Substituting the acrylic cell used here for thinner, more insulating material and insulating the entire device will also reduce this parasitic heat transfer. While the parasitic device heat flux was a minor factor for electrolyte-containing cells, it is more significant for cells containing gelled electrolyte, as discussed above and shown in Fig. 7.

Significantly, while genuine efficiency was consistently lower than estimated efficiency through this work, gelled and partially-gelled electrolytes with small electrode separation values deviated from this trend and offer clear promise for more efficient thermogalvanic devices, particularly when coupled with good cell, device and electrode design. These synergies can now be unambiguously quantified, especially *via* the quantification of total heat flux (as opposed to electrolyte-only heat flux) and this is expected to facilitate total-device optimisation studies.

## Conclusions

This study has demonstrated methodology by which the efficiency of thermogalvanic conversion (of a heat flux through a temperature gradient into electricity) can be unambiguously quantified. This was demonstrated for just the electrolyte, and for the entire assembly; the latter allows ‘total device efficiency’ to be quantified. By comparing the measured efficiency *vs.* the standard route of estimating efficiency, this study shows that they are only comparable for cells with negligible temperature gradients and very small inter-electrode separations. As the temperature gradient increases, the estimated efficiency will overestimate the actual performance by a significant degree; this difference is even more significant with increasing electrode separation, with a complete divergence between estimated and measured efficiency values. Conversely, the measured genuine efficiency of fully-gelled electrolytes can actually exceed the estimated efficiency, but only with small inter-electrode separations, and only if parasitic heat flux through the device apparatus is also discounted. Partially-gelled electrolytes were also identified as the optimum system for efficient thermogalvanic heat-to-electricity conversion, both as an electrolyte and as part of a whole device.

The distinction between absolute efficiency and Carnot efficiency is also important; as the applied temperature difference increases the Carnot efficiency of the thermogalvanic cell decreases slightly, but this is slightly misleading as the absolute overall efficiency actually increases significantly. These observations, combined with this new methodology, will support the rational design of complete thermogalvanic devices, and thus support increasingly efficient waste heat valorisation.

## Data availability

The datasets supporting this article have been uploaded as part of the ESI.†

## Author contributions

Conceptualisation, LA; funding acquisition, LA; investigation, M. A. T., M. A. B. and L. A.; methodology, M. A. T., M. A. B. and L. A.; project administration, LA; supervision, M. A. B. and L. A.; visualisation, L. A.; writing, M. A. T., M. A. B. and L. A.

## Conflicts of interest

There are no conflicts to declare.

## Supplementary Material

SC-013-D1SC06340E-s001
