# Supplementary material for: Direct measurement of the genuine efficiency of thermogalvanic heat-to-electricity conversion in thermocells
Source: Chem Sci. 2022 Apr 5;13(17):4984–98. doi: 10.1039/d1sc06340e (PMC9068204; doi:10.1039/d1sc06340e)
Supplement: SC-013-D1SC06340E-s001 [file SC-013-D1SC06340E-s001.pdf]

Electronic Supplementary Material (ESI) for Chemical Science.
This journal is © The Royal Society of Chemistry 2022

Electronic Supplementary Information for:

Direct measurement of the genuine efficiency of the thermogalvanic heat flux-to-electricity conversion in thermocells

Maria A. Trosheva,[a] Mark A. Buckingham,[a,b] and Leigh Aldous[a]*

[a] Department of Chemistry, Britannia House, King's College London, London, SE1 1DB, UK

[b] Present address: Department of Materials, The University of Manchester, Oxford Road, Manchester, M13 9PL, UK

* Corresponding author: leigh.aldous@kcl.ac.uk

Table of Contents:

Table S1: Summary of experimental data for thermogalvanic cells without the heat flux sensor (page 3)

Table S2: Summary of experimental data for thermogalvanic cells with the heat flux sensor thermally in-series (page 4)

Figure S1: Plots of absolute efficiency and power to support Figures 5 and 6 (page 5)

Figure S2: Plots of the power generated as a function of electrode separation, plotting gelled vs ungelled and with vs without the heat flux sensor thermally in-series (page 6)

Table S3: Summary of experimental data for thermogalvanic cells with and without the heat flux sensor thermally in-series, as a function of gelation and electrode separation (page 7)

Table S1. Summary of the thermogalvanic power measured as a function of electrode separation distance (top) and applied temperature difference (bottom) for the thermogalvanic cell **without** the heat flux sensor in-series; all other details are as per Figure 1.

Experimental Conditions			Thermogalvanic Measurement Results			
d / mm	T_h / °C	T_c / °C	ΔT_{exp} / K	$-V_{ocp}$ / mV	$-j_{sc}$ / A m^{-2}	P_{max} / mW m^{-2}
4.4	40	20	17.8	24.9	15.4	96
9.5			18.0 (± 0.6)	25.3 (± 0.1)	12.3 (± 0.5)	78 (± 5)
18.9			18.3	25.6	9.1	58
29.3			18.2 (± 0.2)	25.4 (± 0.3)	5.8 (± 1.3)	37 (± 8)
39.9			18.2	25.5	4.2	27
29.3	40	20	18.3	25.6	12.2	78
	45	20	22.9	32.1	14.3	115
	50	20	27.4	38.3	18.9	181
	55	20	31.8	44.5	25.0	278
	60	20	36.2	50.7	29.3	371

Key for Tables S1, S2 and S3:

Errors shown as (±) represent the standard deviation of triplicate measurements (Table S1) or between 3 to 5 repeat measurements (Table S2)

d = Electrode separation distance

T_h and T_c = applied temperature to the hot and cold electrodes, respectively

ΔT_{exp} = experienced temperature difference, based upon the V_{ocp}

V_{ocp} = Open circuit potential difference across the hot and cold electrodes

j_{sc} = Steady state short circuit current density

P_{max} = Maximum thermogalvanic power density

p_{max} = Absolute maximum thermogalvanic power generated by the cell

q_{empty} = Steady state, absolute heat flux through the empty cell

q_{total} = Steady state, absolute heat flux through the cell filled with electrolyte

q_m = Measured heat flux, with q_{empty} subtracted from q_{total} to afford the electrolyte-only cell-subtracted heat flux

η_m = Absolute efficiency of thermogalvanic conversion, using measured p_{max} and measured q_m

$\eta_{r,m}$ = Carnot cycle relative measured efficiency of thermogalvanic conversion at the experienced temperatures T_s and T_c, where $T_s = T_h - (T_h - T_c - \Delta T_{exp})$

q_e = Estimated electrolyte-only heat flux using Fourier's Law

η_e = Estimated absolute efficiency of thermogalvanic conversion, using measured p_{max} and estimated q_e

$\eta_{r,e}$ = Estimated Carnot cycle relative efficiency of thermogalvanic conversion, using estimated heat flux and the experienced temperatures T_s and T_c

Table S2. Summary of both the thermogalvanic power and heat flux sensor measurements as a function of electrode separation distance (d, top) and applied temperature difference (ΔT, bottom) for the thermogalvanic cell with the heat flux sensor in-series; all other details are as per Figure 1.

Experimental Conditions			Thermogalvanic Measurement Results					Heat Flux Sensor Measurement Results			Measured Efficiencies		Estimated Heat Flux and Estimated Efficiencies		
d / mm	T_h / °C	T_c / °C	ΔT_{exp} / K	$-V_{ocp}$ / mV	$-j_{sc}$ / A m^{-2}	P_{max} / mWm^{-2}	p_{max} / µW	q_{empty} / mW	q_{total} / mW	q_m / mW	η_m / 10^{-3} %	$\eta_{r,m}$ / %	q_e / mW	η_e / 10^{-3} %	$\eta_{r,e}$ / %
4.4			15.3	21.5	14.2	76	7.1	73	312	239	3.0	0.060	180	4.0	0.08
9.5			16.1 (± 0.7)	22.5 (± 0.9)	11.4 (± 0.3)	65 (± 2)	6.1 (± 0.1)	68 (± 12)	316 (± 21)	248 (± 24)	2.4 (± 0.2)	0.047 (± 0.005)	87.5 (± 3.6)	6.9 (± 0.3)	0.13 (± 0.1)
18.9	40	20	16.3	22.9	7.2	41	3.9	49	317	268	1.4	0.027	44.8	8.7	0.16
29.3			16.7 (± 0.7)	23.4 (± 0.1)	5.6 (± 0.4)	33 (± 3)	3.1 (± 0.3)	36 (± 4)	272 (± 17)	236 (± 17)	1.3 (± 0.2)	0.024 (± 0.003)	29.6 (± 1.2)	10.4 (± 0.1)	0.19 (± 0.2)
39.9			16.0	22.3	4.3	24	2.3	22	281	259	0.9	0.017	20.7	11.0	0.21
	40	20	16.1	22.5	11.4	65	6.1	68	316	248	2.4	0.047	87.5	6.9	0.13
	45	20	19.7	27.6	14.7	101	9.5	91	385	295	3.2	0.051	107	8.9	0.14
9.5	50	20	23.5	32.9	17.9	147	13.8	110	478	368	3.7	0.050	128	10.8	0.15
	55	20	27.3	38.2	20.7	197	18.6	128	567	439	4.2	0.050	149	12.5	0.15
	60	20	30.1	42.2	26.6	280	26.4	145	729	584	4.5	0.048	164	16.1	0.17

Figure S1. Plots of (a) the absolute thermogalvanic conversion efficiency as a function of electrode separation (Carnot-relative efficiency shown in Figure 5(c)); (b) thermogalvanic power generated with and without the heat flux sensor in-series *vs* the experienced temperature difference, ΔT_{exp} (the same power plotted *vs* the applied temperature difference, ΔT_{app}, is shown in Figure 6(a)), and (c) the absolute thermogalvanic conversion efficiency as a function of ΔT_{app} (Carnot-relative efficiency shown in Figure 6(d)). Please refer to Figures 5 and 6 for full experimental details.

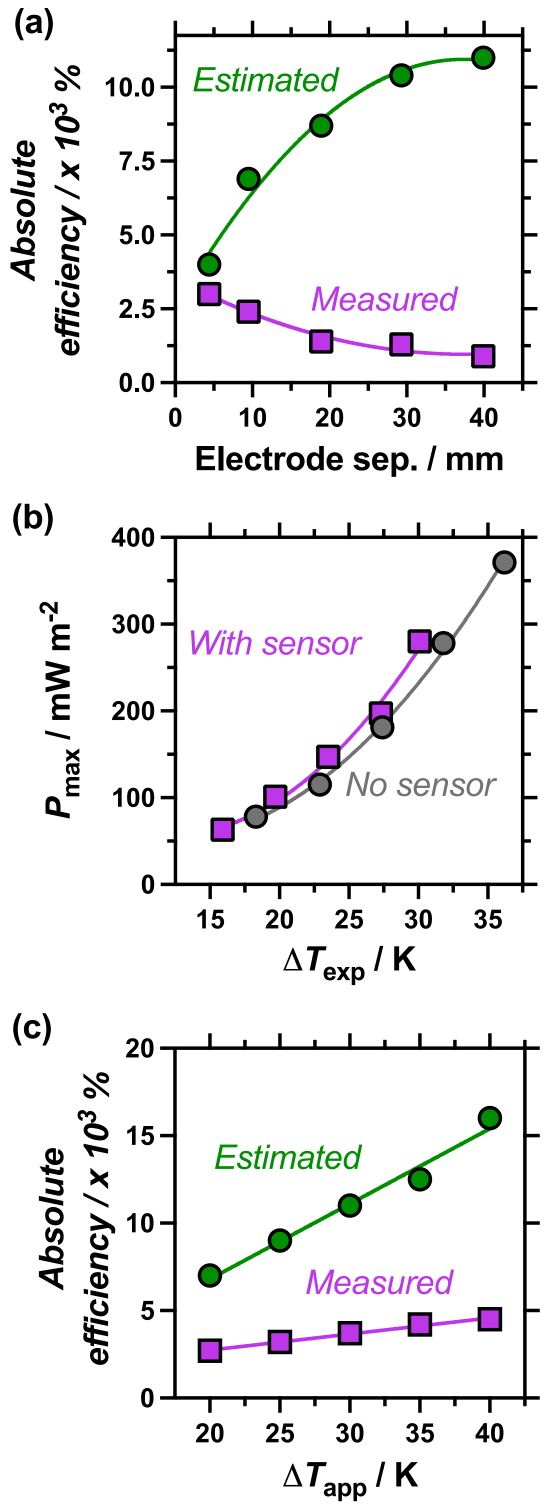

Figure S2. Plot of the thermogalvanic power generated by the as-prepared 0.4 M $K_3/K_4[Fe(CN)_6]$ at ΔT_{app} = 20 K, using partially filled 3-sided cells (see Experimental for full details). They were measured with (purple) and without (grey) the heat flux sensor in-series, either with 3 wt% eq. of sodium acrylate powder added (labelled as gelled; hexagon and diamond) or prior to the addition of the sodium acrylate powder (circle and square). Reported as a function of electrode separation (13.6, 20.9 and 29.2 mm).

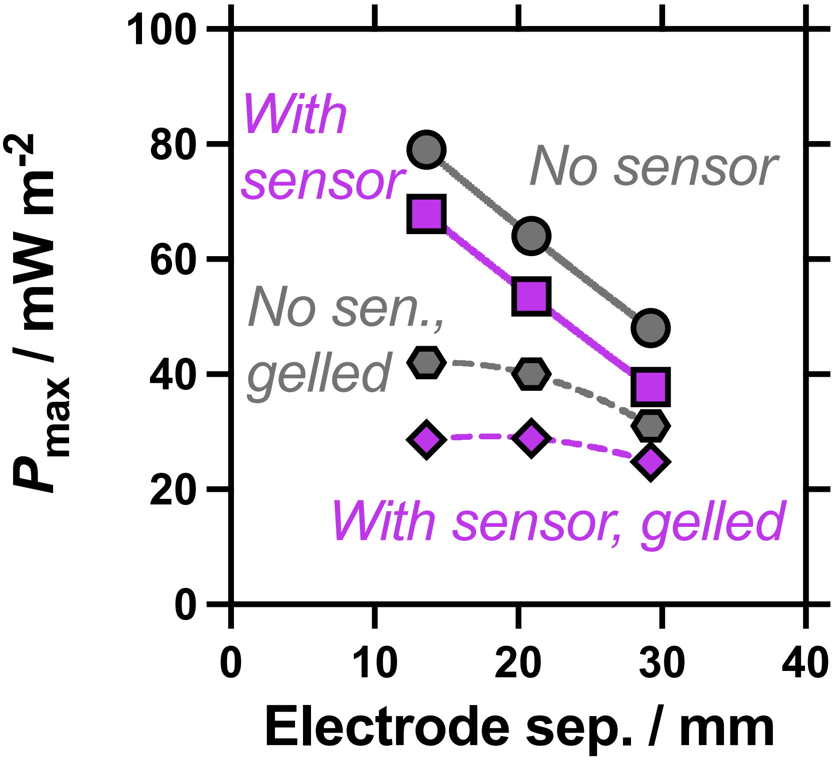

Table S3. Summary of the thermogalvanic and heat flux sensor measurements, investigated as a function of electrode separation distance (*d*) and the weight percent equivalent of sodium polyacrlytate powder added to the cell as a gelling agent. Measurements were performed with heat flux sensor in series (bottom) or without the heat flux sensor present (top). Measurements were performed in a 3-sided cell with the cell partially filled, as detailed in the Experimental section. All values are the average of between 2 to 4 repeat measurements.

Experimental Conditions		Thermogalvanic Measurement Results					Heat Flux Sensor Measurement Results			Measured Efficiencies		Estimated Heat Flux and Estimated Efficiencies		
d / mm	Gel / wt%	ΔT_{exp} / K	$-V_{ocp}$ / mV	$-j_{sc}$ / A m^{-2}	P_{max} / mW m^{-2}	p_{max} / µW	q_{empty} / mW	q_{total} / mW	q_{m} / mW	η_{m} / 10^{-3} %	$\eta_{r,m}$ / %	q_{e} / mW	η_{e} / 10^{-3} %	$\eta_{r,e}$ / %
13.6	0	18.4	25.7	12.3	79	5.6	n/a	n/a	n/a	n/a	n/a	n/a	n/a	n/a
	3	19.7	27.6	6.5	42	3.2								
20.9	0	18.4	25.8	9.9	64	4.6	n/a	n/a	n/a	n/a	n/a	n/a	n/a	n/a
	3	19.5	27.3	5.9	40	2.9								
29.2	0	18.5	25.9	7.4	48	3.5	n/a	n/a	n/a	n/a	n/a	n/a	n/a	n/a
	3	19.4	27.2	4.5	31	2.2								
13.6	0	17.1	23.9	11.4	67.9	4.8	72	186	114	4.2	0.077	49	9.8	0.179
	1.5	18.6	26.1	10.1	65.8	4.7	72	101	29	16.1	0.269	54	8.7	0.146
	3	18.2	25.5	4.5	28.6	2.0	72	90	18	11.6	0.197	53	3.9	0.066
20.9	0	17.0	23.8	9.0	53.5	3.8	73	197	124	3.1	0.056	32	11.9	0.218
	3	18.9	26.5	4.4	28.9	2.1	73	97	25	8.3	0.137	35	5.8	0.096
29.2	0	16.6	23.2	6.5	37.8	2.8	75	206	131	2.1	0.039	23	12.1	0.226
	3	18.9	26.4	3.8	24.8	1.8	75	121	46	3.9	0.065	26	7.0	0.116
